# *Tomato chlorosis virus* CPm protein is a pathogenicity determinant and suppresses host local RNA silencing induced by single-stranded RNA

**DOI:** 10.3389/fmicb.2023.1151747

**Published:** 2023-03-28

**Authors:** Xiao Yang, Xiangwen Luo, Yu Zhang, Zhanhong Zhang, Xian OuYang, Xiaobin Shi, Xiaoyuan Lv, Fan Li, Songbai Zhang, Yong Liu, Deyong Zhang

**Affiliations:** ^1^Longping Branch, College of Biology, Hunan University, Changsha, Hunan, China; ^2^Key Laboratory of Pest Management of Horticultural Crop of Hunan Province, Hunan Plant Protection Institute, Hunan Academy of Agricultural Science, Changsha, Hunan, China; ^3^Technical Center of Changsha Customs, Changsha, Hunan, China; ^4^College of Plant Protection, Yunnan Agricultural University, Kunming, China

**Keywords:** *Tomato chlorosis virus*, CPm protein, pathogenicity determinant, RNA silencing suppressor, posttranscriptional gene silencing

## Abstract

**Introduction:**

*Tomato chlorosis viru*s (ToCV) is a typical member of the genus *Crinivirus*, which severely threatens *Solanaceae* crops worldwide. The CPm protein encoded by ToCV has been reported to be associated with virus transmission by vectors and is involved in RNA silencing suppression, while the mechanisms remain ambiguous.

**Methods:**

Here, ToCV *CPm* was ectopically expressed by a *Potato virus X* (PVX) vector and infiltrated into *Nicotiana benthamiana* wild-type and GFP-transgenic16c plants.

**Results:**

The phylogenetic analysis showed that the CPm proteins encoded by criniviruses were distinctly divergent in amino acid sequences and predicted conserved domains, and the ToCV CPm protein possesses a conserved domain homologous to the TIGR02569 family protein, which does not occur in other criniviruses. Ectopic expression of ToCV *CPm* using a PVX vector resulted in severe mosaic symptoms followed by a hypersensitive-like response in *N. benthamiana*. Furthermore, agroinfiltration assays in *N. benthamiana* wilt type or GFP-transgenic 16c indicated that ToCV CPm protein effectively suppressed local RNA silencing induced by single-stranded but not double-stranded RNA, which probably resulted from the activity of binding double-stranded but not single-stranded RNA by ToCV CPm protein.

**Conclusion:**

Taken together, the results of this study suggest that the ToCV CPm protein possesses the dual activities of pathogenicity and RNA silencing, which might inhibit host post-transcriptional gene silencing (PTGS)-mediated resistance and is pivotal in the primary process of ToCV infecting hosts.

## 1. Introduction

Plant viruses belonging to the family *Closteroviridae* are divided into four genera: *Ampelovirus, Closterovirus*, *Crinivirus*, and *Velarivirus* (Fiallo-Olive and Navas-Castillo, 2019). Criniviruses are restricted to the phloem and rely on whitefly vectors of the genera *Bemisia* and/or *Trialeurodes* for plant-to-plant transmission (Kiss et al., 2013). *Tomato chlorosis virus* (ToCV) is a typical member of the genus *Crinivirus*, which is transmitted in nature by three whitefly species (*Bemisia tabaci*, *Trialeurodes vapourariorum,* and *T. abutilonea*) in a semipersistent manner (Wisler et al., 1998). ToCV was documented to cause severe yields and economic losses to *Solanaceae* crops worldwide, probably rapidly dispersing along with its vector of Q-biotype whitefly (*Bemisia tabaci* Q-biotype) (Huang et al., 2021). The genome of ToCV comprises two single-stranded and positive-sense RNA segments, namely, RNA1 (8593–8,596 nt) and RNA2 (8242–8,247 nt) (Wintermantel et al., 2005; Lozano et al., 2006, 2007). RNA1 contains four overlapping open reading frames (ORF 1a/1b to ORF3), which encode four proteins associated with virus replication and the suppression of gene silencing, and RNA2 contains nine ORFs (ORF4 to ORF12)-encoded proteins putatively involved in virus encapsidation, cell-to-cell movement, whitefly transmission, and the suppression of gene silencing (Orilio et al., 2014; Wintermantel, 2018). Most proteins encoded by ToCV were not experimentally validated.

The CPm protein encoded by criniviruses possessed multiple functions in previous documents. CPm protein of the typical member in the genus *Crinivirus*, *Lettuce infectious yellows virus* (LIYV), constitutes an integral part of the tail protein complex, whereby CPm binds to the anterior foregut of whitefly epithelial cells for plant-to-plant transmission (Stewart et al., 2010; Chen et al., 2011). CPm protein encoded by *Beet yellows closterovirus* (BYV) is a prerequisite for virus cell-to-cell movement in the host (Alzhanova et al., 2000). CPm encoded by ToCV was verified to accentuate the ectopical virus *Potato virus X* (PVX)-infected host and plausibly possessed silencing suppression activity (Canizares et al., 2008), but further verification is needed, and the mechanisms remain ambiguous.

RNA silencing-mediated resistance is critical for plants to prevent viral infection. To counteract antiviral RNA silencing in the host, plant viruses have developed an effective strategy to encode RNA silencing suppressor (RSS) proteins (Hirai et al., 2008). RNA silencing is induced by single- and/or double-stranded RNA, which is integrated with host factors, such as Dicer (or DCL for Dicer-like), to incorporate into an RNA-induced silencing complex (RISC) to degrade target viral RNAs (Hamilton and Baulcombe, 1999). In plants, RNA silencing is likely induced at the single-cell level of virus infection, and generally, the mobile silencing signal would be generated simultaneously, which could move from cell to cell through plasmodesmata and systemically *via* the vascular system to induce systematic RNA silencing in plants (Voinnet and Baulcombe, 1997; Himber et al., 2003). The RSS proteins encoded by plant viruses were demonstrated to have great diversity in sequence and structure, which could prevent RNA silencing by distinct strategies (Musidlak et al., 2017). Therefore, further studies to identify new RNA silencing suppressors and their modes of action would provide a better understanding of the molecular mechanisms by which viruses prevent the host resistance induced by RNA silencing (Voinnet, 2005; Diaz-Pendon and Ding, 2008).

It is well documented that the RSS proteins of plant viruses also exhibit properties such as the enhancement of viral accumulation and pathogenicity (Peretz et al., 2007; Zhu et al., 2012). This dual activity of RSS proteins hinted at a probable intrinsic relationship between viral pathogenesis and RNA silencing suppression, which would be pivotal for virus infecting hosts (Silhavy and Burgyan, 2004; Voinnet, 2005).

The CPm protein of ToCV was previously verified to be activated to accentuate PVX infection and to plausibly suppress host RNA silencing, reminisced of the dual activity of RSS proteins of plant viruses. However, the molecular mechanisms by which the CPm protein of ToCV activates this dual activity are largely unknown. In this study, a detailed characterization of the CPm protein encoded by the ToCV Shangdong isolate (ToCV-SDSG, GenBank: AGN91010.1) in terms of its dual activities of pathogenicity and suppression of PTGS was deciphered. The results described in this paper provide novel insight to better understand the interaction between criniviruses and their host plants.

## 2. Materials and methods

### 2.1. Plant material

Wild-type and transgenic GFP 16c of *Nicotiana benthamiana* were grown in pots with peat and vermiculite (3:1, w/w) inside a culture room with 25 ± 2°C and 60% relative humidity with 16-h light/8-h dark illumination. The third or fourth leaves of 4- to 6-week-old *N. benthamiana* plants were used for infiltration with an *Agrobacterium* culture.

### 2.2. Sequence analysis

The amino acid sequences of ToCV and 11 other crinivirus CPm proteins were retrieved from GenBank.^1^ Multiple sequence alignment analysis was performed using DNAStar software v.7.1 with the ClustalW alignment method. Phylogenetic analysis was performed by MEGA 7.0 using the neighbour-joining method with 1,000 bootstrap replications. GenBank accession numbers are shown in the trees. The conserved domains of CPm proteins were identified using the Conserved Domain Database (CDD)^2^ in GenBank.

### 2.3. Plasmid construction

The complete sequence of ToCV *CPm* was cloned using specific primers containing the adapter of homologous recombination (Supplementary Table S1) from the pGEMT::CPm vector with full-length ToCV *CPm*, which had been verified by Sanger sequencing and kept in our lab. ToCV CPm was inserted into pGR106 (a *Potato virus X*-based vector) for pathogenicity experiments, into pBIN-3HA for RNA silencing analysis, and into pET28α from prokaryotes expressing and purified the recombinant CPm protein. The vectors expressing the *βC1* gene of *Tomato yellow leaf curl China virus* beta-satellite (TYLCCNB) and the *P19* gene of *Tomato bushy stunt virus* (TBSV) were kindly gifted by Prof Zhenghe Li (Zhejiang University, Hangzhou, China) and described previously (Chu et al., 2000; Zhou et al., 2021). All plasmids in this study were constructed by homologous recombination and transformed into *A. tumefaciens* GV3101 or *Escherichia coli* DH5α (Gong et al., 2018). The primers for gene cloning and vector construction are listed in Supplementary Table S1.

### 2.4. Protein extraction and western blot analysis

The protein was extracted from inoculated or systemic leaves using a protein extraction buffer [1% SDS, 2 mM EDTA, 10 mM Tris (pH 7.5)]. Ten micrograms of total protein was separated by 10% sodium dodecyl sulfate–polyacrylamide gel electrophoresis (SDS–PAGE) and transferred to a polyvinylidene difluoride (PVDF) membrane. The PVDF membrane was blocked with either 5% nonfat dry milk in TBS with 0.1% Tween-20 for 1 h at room temperature and then incubated for 1 h at room temperature with primary mouse monoclonal antibody [green fluorescent protein (GFP) or PVX CP protein monoclonal antibody, HuaAn Biotech, Beijing, China], followed by goat anti-mouse secondary antibody conjugated to horseradish peroxidase (Bio-Rad). Blotted membranes were washed thoroughly and visualized by chemiluminescence mode (Azure C300, GE, United States).

### 2.5. Total RNA extraction and RT–qPCR analysis

Total RNA was extracted from the inoculated or systemic leaves using TRIzol reagent (Invitrogen, Thermo Fisher Scientific, Beijing, China). The concentration and purity of extracted RNA were assayed by a NanoDrop 2000 (Thermo Fisher Scientific, Beijing, China), and 1,000 ng of RNA was used to synthesize the first strand of cDNA for qRT–PCR using a TransScript All-in-One First-Strand cDNA synthesis SuperMix for qPCR (One-Step gDNA Removal) kit (TransGen Biotech, Beijing, China) following the manufacturer’s instructions. qPCR was conducted using the TransStart Green qPCR SuperMix UDG kit (TransGen Biotech, Beijing, China) on the qTOWER3G qPCR system (Analytik Jena, Jena, Germany). The *NbActin* gene (GenBank: JQ256516.1) was selected as an internal control for the assays. The primers for qPCR are listed in Supplementary Table S1. All the gene expression data were collected in three technical replicates for each biological condition tested to assess reproducibility, and the relative gene expression levels were calculated using the 2^−ΔΔCt^ method for analysis.

### 2.6. Electrophoretic mobility shift assays for RNA–protein complexes

The 5′-biotinylated double-stranded and single-stranded GFP RNA probes were obtained from Sangon Biotech (Shanghai, China) and is listed in Supplementary Table S2. The 5′-biotinylated double-stranded and single-stranded GFP RNA probes (100 pM) were denatured at 95°C for 2 min. The recombinant His-CPm protein used in this study was purified from a prokaryotic expression system according to manufacturer’s instructions (E8200S, New England Biolabs). The RNA probes were incubated with the purified protein at room temperature for 30 min in a final volume of 20 μl, supplemented with RNAase inhibitor. The entire reaction mixture was run on a nondenaturing 0.5× TBE 6% polyacrylamide gel for 1 h at 60 V at 4°C and then transferred onto Biodyne® B nylon membranes (Pall Corporation, Shanghai, China) using the semi-dry transfer system (Apelex) in pre-cooled 0.5x TBE buffer. Labeled dsRNA GFP probe was set as negative control, amd CAP protein of *Escherichia coli* co-incubated with the 5′-biotinylated double-stranded GFP RNA probe was set as positive control (Liu-Johnson et al., 1986). Signals were visualized with reagents included in the kit and ChemiDoc XRS (Bio-Rad Laboratories, United States) (Hellman and Fried, 2007; Fillebeen et al., 2014).

## 3. Results

### 3.1. Phylogenetic analyses and domain architecture of ToCV-encoded CPm protein

**Figure 1:**
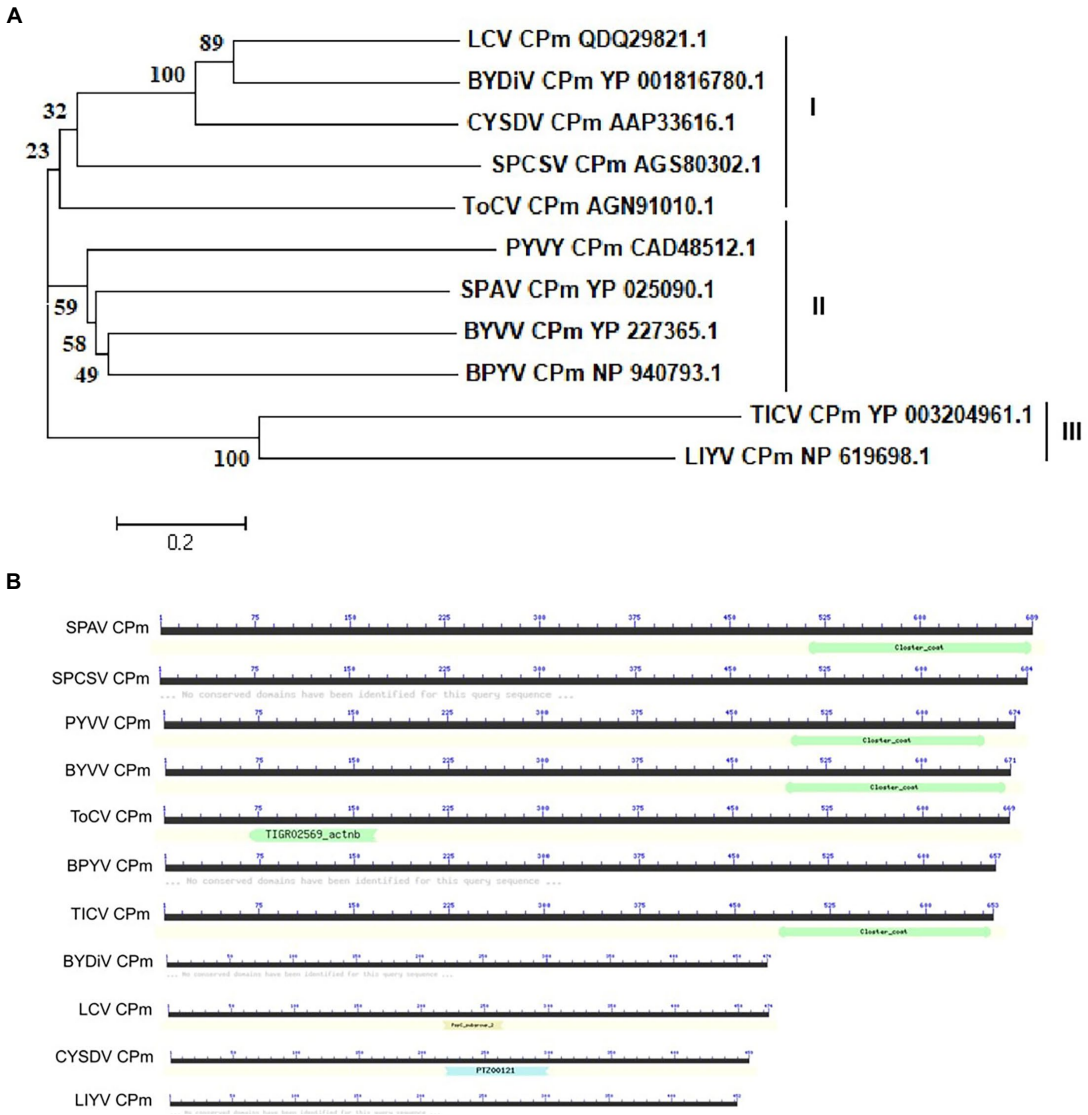
Phylogenetic relationships and domain structure of CPm amino-acid sequences of representative criniviruses. **(A)** The amino-acid sequences of the CPm from 11 criniviruses were aligned using the neighbor-joining method with the MEGA v7.0 program with 1,000 replications. The numbers beside each node represent the percentages for the bootstrap value. The protein accession number in Genbank were shown following virus name. BPYV, *Beet psedoyellows virus*; BYDiV, Bean yellow disorder virus; BYVV, Blackberry yellow vein-associated virus; CYSDV, *Cucurbit yellow stunting disorder virus*; LCV, Lettuce chlorosis virus; LIYV, *Lettuce infectious yellows virus*; PYVV, Potato yellow vein virus; SPAV, *Strawberry pollidosis-associated virus*; SPCSV, Sweet potato chlorotic stunt virus; TICV, Tomato infectious chlorosis virus; ToCV, *Tomoto chlorosis virus*; **(B)** The conserved peptidase domain was identified using the Conserved Domain Database (CDD).

The CPm protein of ToCV (Shagndong isolate, GenBank: AGN91010.1) encodes a protein of 669 amino acids (aa). To examine the evolutionary relationships of CPm sequences from different criniviruses, we first examined the phylogenetic dendrogram of the complete CPm amino acid sequences of 11 different criniviruses. As shown in Figure 1, the phylogenetics, structure and length were distinctly different in the crinivirus proteins. Phylogenetic analysis of amino acid sequences of CPm proteins supported three subgroup clusters, and ToCV CPm protein belonged to subgroup I with *Lettuce chlorosis virus* (LCV), *Bean yellow disorder virus* (BYDiV), *Cucurbit yellow stunting disorder virus* (CYSDV), and *Sweet potato chlorotic stunt virus* (SPCSV) (Figure 1A). Otherwise, the length of CPm proteins in the subgroup differed, with the longest being 684 amino acids for the SPCSV CPm protein and the shortest being 474 amino acids for the LCV CPm protein (Figure 1B). Figure 1B also shows the differences in structure in CPm proteins. The ToCV-encoded CPm protein contains a domain homology to the TIGR02569 family protein, which has only been discovered in actinobacteria thus far (Dong et al., 2019), but lacks the closter_coat domain, which a few CPm proteins of criniviruses possess (Figure 1B). The distinct differences probably inferred the different functions of CPm proteins in crinivirus infection, and discovering the novel functions of CPm proteins of these criniviruses would provide more deep insights into the interaction of criniviruses with hosts.

### 3.2. *Tomato chlorosis viru*s CPm protein is a symptom determinant and induces H_2_O_2_ accumulation in *Nicotiana benthamiana*

**Figure 2:**
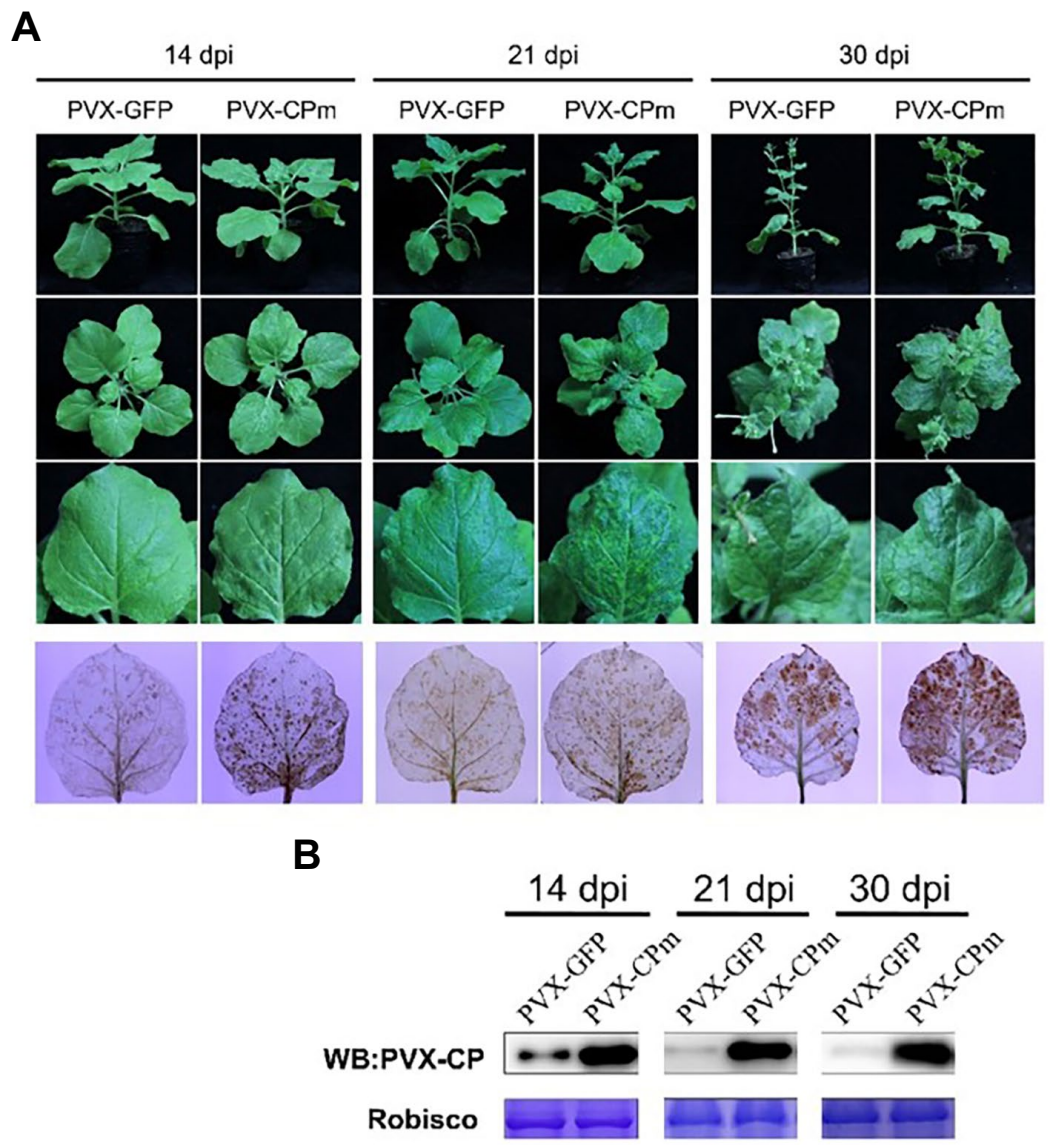
Symptoms and H_2_O_2_ accumulation exhibited in plants inoculated with potato virus X (PVX) or PVX-CPm. **(A)** Severe mosaic symptoms induced H_2_O_2_ accumulation in leaves of *Nicotiana benthamiana* plants by PVX-CPm. The 3rd line of infected leaves were stained with 3,3′-diaminobenzidine (DAB) and photographed directly. **(B)** Western blotting analysis of PVX CP protein in systemic leaves from *N. benthamiana* plants infected with PVX-GFP or PVX-CPm.

The symptoms of interveinal yellowing, thickening, and bronzing were displayed in older leaves in ToCV-infected plants, which causes severe yield loss and commodity reduction in some of the *Solenacea*e crops (Fiallo-Olive and Navas-Castillo, 2019; Liu et al., 2021). To determine whether the CPm protein plays a key role in pathogenicity during ToCV infection of the host, the *CPm* gene was introduced into pGR106, a *Potato virus X* (PVX)-based vector, for overexpression. *A. tumefaciens* carrying PVX-CPm was inoculated into young *N. benthamiana* plants at the four-week stage by agroinfiltration. During the first 14 dpi, the phenotype of *N. benthamiana* inoculated with PVX-CPm was similar to that of the plants inoculated with PVX-GFP. And PVX-GFP -infected plants showed signs of recovery, with a loss of veinal chlorosis and mosaic symptoms by 30 dpi. Otherwise, PVX-CPm-infected plants and systematic leaves showed more veinal chlorosis and mosaic symptoms than PVX-GFP (control group) at 14 and 30 dpi (the 1st to 3rd line in Figure 2A).

To determine whether the symptoms were attributed to the accumulation of H_2_O_2_, PVX-GFP- and PVX-CPm-infected leaf tissues were analyzed by the 3,3′-diaminobenzidine (DAB) uptake method. In the presence of H_2_O_2_, DAB polymerizes to produce a deep brown color that can be visualized after ethanol clearing of the tissues (Sharma and Ikegami, 2010). As shown in the 4th line in Figure 2A, obviously higher concentrations of H_2_O_2_ accumulated in systemically infected leaves of PVX-CPm-infected plants than in those of PVX-GFP-infected plants. To determine whether severe symptoms and higher concentrations of H_2_O_2_ accumulation in the presence of CPm protein resulted from higher accumulation of PVX, western blotting against PVX CP protein was employed to examine the accumulation of PVX CP protein (Figure 2B). More PVX CP proteins accumulated in PVX-CPm-infected plants than in PVX-GFP-infected plants at 14–30 dpi, suggesting that CPm protein is a probable virulence factor that enhances replication of PVX in *N. benthamiana.* Taken together, these data inferred that ToCV CPm protein is a symptom determinant that can elicit H_2_O_2_ accumulation when expressed from PVX-CPm.

### 3.3. *Tomato chlorosis viru*s CPm protein induces an endoplasmic reticulum stress response in *Nicotiana benthamiana*

**Figure 3:**
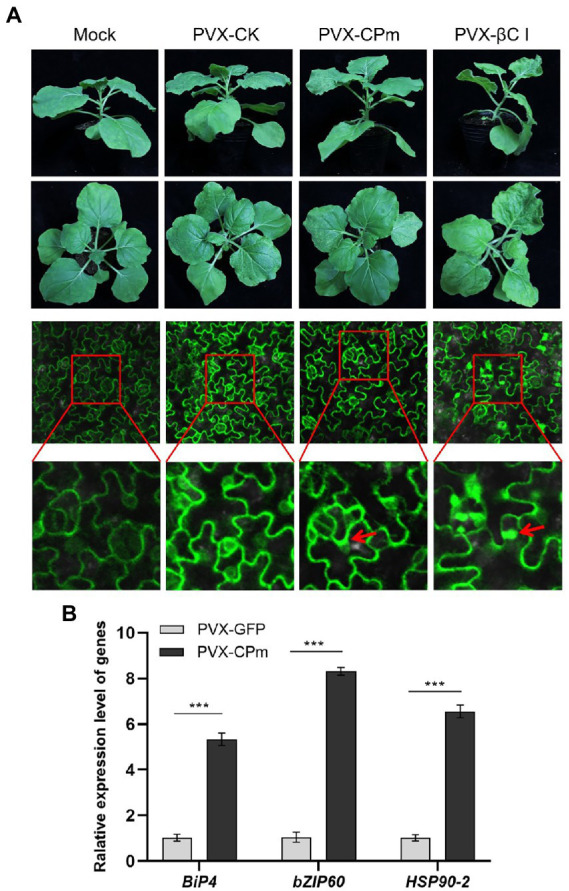
Endoplasmic reticulum (ER)-body and relative mRNA levels of the indicated ER-stress marker genes in *CPm*-expressing *Nicotiana benthamiana* 16c plants. **(A)** Symptoms and ER-body induced in leaves of *N. benthamiana* 16c plants induced by PVX-CPm. **(B)** Relative mRNA levels of *ER-localized binding* (*Bip*), *Heat shock 90–2* (*HSP90-2*), and *basic leucinezipper 60* (*bZIP60*) were measured in *Potato virus X* (PVX)-CPm and PVX-GFP vector-infected *N. benthamiana* 16c plants at 14 days post inoculation (dpi) by RT-qPCR. Values are the mean ± SD. Highly significant differences (***ρ < 0.001) between samples in each pair are indicated.

To investigate whether the ToCV CPm protein induces an ER stress response in *N. benthamiana*-infected leaves. GFP-transgenic *N. benthamiana* 16c leaves were inoculated with *A. tumefaciens* of PVX-CPm, PVX-βC1 (a positive control), or PVX-CK (PVX-GFP as a negative control). As shown in the 1st and 2nd lines of Figure 3A, PVX-CPm induced severe symptoms in the systematic leaves of *N. benthamiana* 16c plants similar to PVX-CPm infected wild-type *N. benthamiana* plants (Figure 2A). Otherwise, the symptoms in the systematic leaves of *N. benthamiana* 16c plants induced by PVX-CPm were less than *N. benthamiana* 16c plants induced by PVX-βC1 (Figure 3A). These results might reinforce the notion that the ToCV CPm protein is a pathogenicity determinant (Figure 2). The results of the ER body and relative mRNA levels of the indicated ER stress marker genes in CPm-expressing *N. benthamiana* 16c plants confirmed that the ToCV CPm protein induced more ER bodies and relatively higher expression of all selected ER stress marker genes, including *ER-localized binding* (*Bip*), *heat shock 90–2* (*HSP90-2*), and *basic leucine zipper 60* (*bZIP60*) (the 3rd and 4th lines in Figures 3A,B). These results suggested that the CPm protein induces ER stress.

### 3.4. *Tomato chlorosis viru*s CPm protein blocks local RNA silencing triggered by single-stranded GFP but not by double-stranded GFP

**Figure 4:**
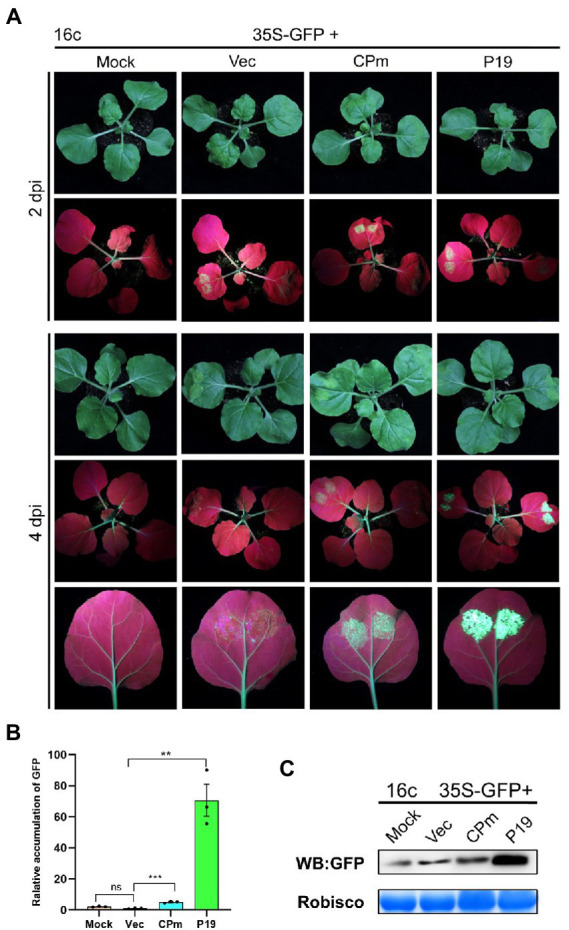
*Tomato chlorosis viru*s CPm protein suppressing local RNA silencing triggered by GFP ssRNA. **(A)** Photographs taken under the white light and UV light of *Nicotiana benthamiana*16c leaves at 2 and 4 days post-infiltration (dpi) with *Agrobacterium tumefaciens* harboring 35S-GFP, either in combination with the pBIN19-3HA empty vector (Vec), or with constructs expressing *CPm* or *P19*. **(B)** Relative mRNA levels of GFP were measured by RT-qPCR. **(C)** Western blotting analysis of GFP protein extracted from the zones infiltrated with *Agrobacterium tumefaciens* harboring the constructs indicated above each lane at 4 dpi.

Previous studies demonstrated that the pathogenicity determinant proteins encoded by plant viruses were also correlated with the function of RNA silencing (Li et al., 2015), and the ToCV CPm protein was documented to plausibly suppress local ssRNA-induced RNA silencing in plants. To test whether ToCV CPm suppresses systematic ssRNA- and dsRNA-induced RNA silencing, *N. benthamiana* wild type or 16c was inoculated into *A. tumefaciens* containing a construct capable of expressing *CPm* from the CaMV 35S promoter pBIN-3HA. As shown in Figure 4A, co-infiltration of 35S-GFP with the vector expressing *CPm* in *N. benthamiana* 16c, a fluorescence that lasted for at least 4 days, was observed in the infiltrated area, whereas no such fluorescence was observed for coinoculations with the empty vector, and a strong fluorescence appeared for coinoculations with the vector expressing *P19* (Figure 4A). Western blotting and RT-qPCR also verified that the GFP accumulated in cells expressing *CPm*, and more GFP accumulated in cells expressing *P19* than in cells expressing *CPm* (Figures 4B,C). These results inferred that CPm protein blocked the RNA silencing triggered by ssRNA, which was weaker than P19 protein.

**Figure 5:**
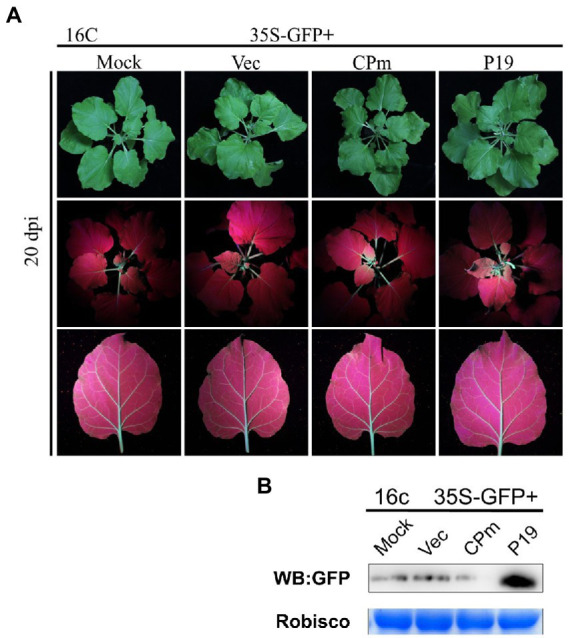
*Tomato chlorosis viru*s CPm protein not suppressing systematic RNA silencing triggered by GFP dsRNA. **(A)** Photographs taken under the white and UV light of *Nicotiana benthamiana*16c leaves at 20 days post-infiltration (dpi) with *Agrobacterium tumefaciens* harboring 35S-GFP, either in combination with the pBIN19-3HA empty vector (Vec), or with constructs expressing *CPm* or *P19*. **(B)** Western blotting analysis of GFP protein extracted from the zones infiltrated with *Agrobacterium tumefaciens* harboring the constructs indicated above each lane at 20 dpi.

To test whether the signals of CPm protein suppressing the RNA silencing triggered by ssRNA were systematically transmitted, GFP expression was detected in systematic leaves at 20 dpi. There was no fluorescence displayed under UV light (Figure 5A), and the GFP did not accumulate in cells expressing *CPm* but accumulated in cells expressing *P19* (Figure 5B). These results suggested that the CPm protein did not suppress systematic RNA silencing.

**Figure 6:**
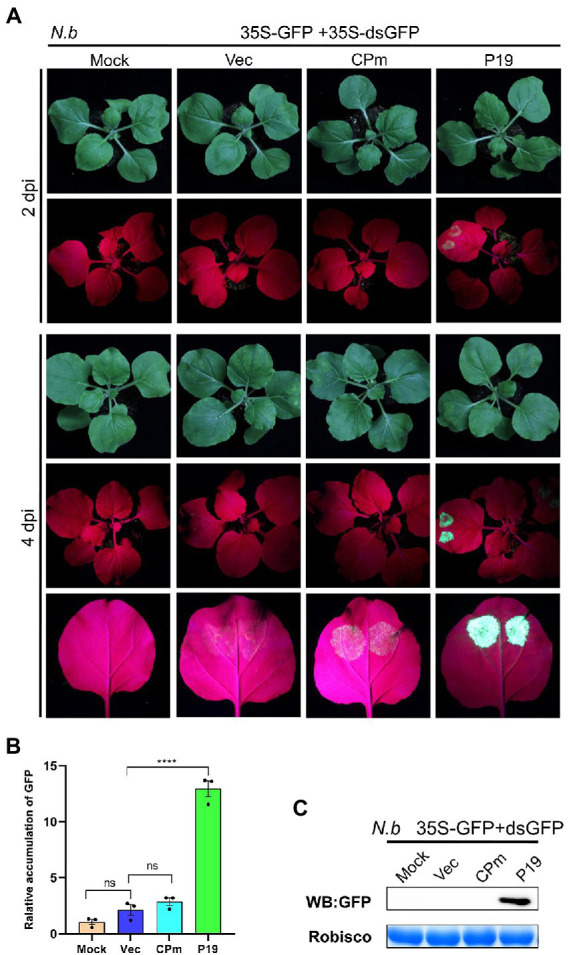
*Tomato chlorosis viru*s CPm not suppressing RNA silencing triggered by GFP dsRNA. **(A)** Photographs taken under the white and UV light of *Nicotiana benthamiana* leaves at 2 and 4 days postinfiltration (dpi) with *A. tumefaciens* harboring 35S-GFP and 35S GF-IR, either in combination with the pBIN19-3HA empty vector (Vec), or with constructs expressing *CPm* or *P19*. **(B)** Relative mRNA levels of GFP were measured by RT-qPCR. **(C)** Western blotting analysis of GFP protein extracted from the zones infiltrated with *Agrobacterium tumefaciens* harboring the constructs indicated above each lane at 4 dpi.

To test whether CPm protein suppresses the RNA silencing triggered by dsRNA, coinfiltration of 35S-GFP and 35S-dsGFP, which express the dsRNA of the reporter gene *GFP*, with the vector expressing *CPm* was performed in wild-type *N. benthamiana*. Bright fluorescence emerged in the leaves of wild-type *N. benthamiana* expressing *P19* at 2 and 4 dpi; in contrast, there was no fluorescence for expressing *CPm* (Figure 6A). Western blotting and RT-qPCR also verified that the GFP was distinctly accumulated for expressing *P19* but not for expressing *CPm* (Figures 6B,C). These results showed that the CPm protein did not suppress RNA silencing induced by dsRNA.

### 3.5. *Tomato chlorosis viru*s CPm protein binds double-stranded RNA

**Figure 7:**
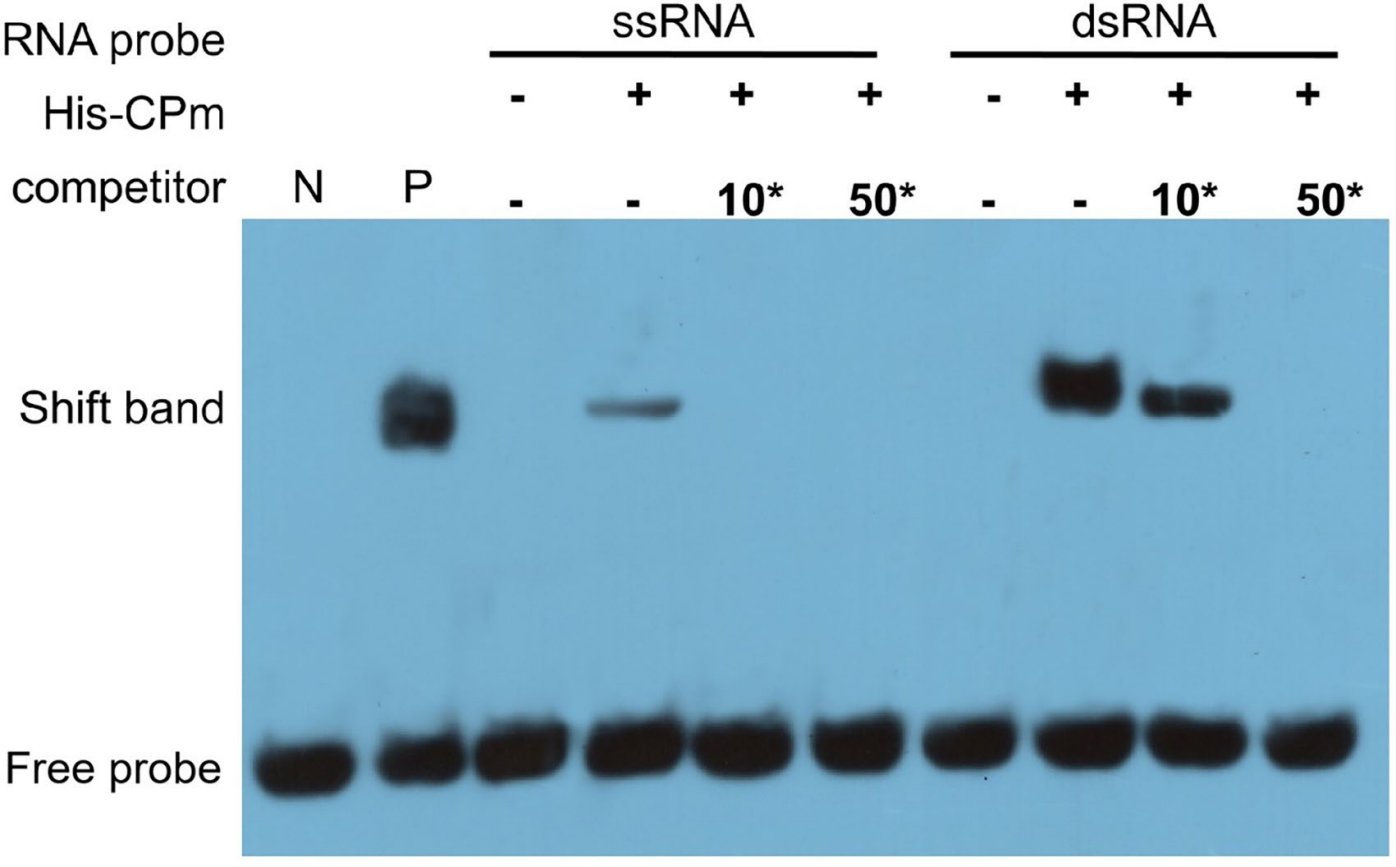
*Tomato chlorosis viru*s CPm binding the dsRNA, but not the ssRNA of GFP by electrophoretic mobility shift assay (EMSA). 10*, 10-fold dilution of probe; 50*, 50-fold dilution of probe. N, labeled dsRNA GFP probe was set as negative control; P, CAP protein of *Escherichia coli* co-incubated with the 5′-biotinylated double-stranded GFP RNA probe was set as positive control.

It is well documented that RNA silencing in plants can be triggered by ssRNA and dsRNA (Hamilton and Baulcombe, 1999), and ToCV CPm protein only suppresses local ssRNA-induced RNA silencing (Figure 4). To test whether CPm protein can directly sequester dsRNA to quench dsRNA-triggered silencing, an electrophoretic mobility shift assay (EMSA) was employed to test CPm protein interactions with ssRNA and dsRNA. As expected, His-tagged purified ToCV CPm protein competitively bound the 5′-biotinylated double-stranded GFP RNA but not the 5′-biotinylated single-stranded GFP RNA (Figure 7). This result suggested that the dsRNA binding activity of CPm protein is probably correlated with its activity of suppressing local RNA silencing induced by ssRNA.

## 4. Discussion

ToCV is one of the 14 accepted species in the genus *Crinivirus*, one of the four genera in the family Closteroviridae (Fiallo-Olive and Navas-Castillo, 2019). The World Tomato Processing Council (WPTC) reported that tomato is the largest vegetable crop worldwide but is easily threatened by ToCV (Kaur et al., 2017; Moodley et al., 2019). An increasing number of documents recorded the epidemic and severe commodity loss in tomato and other solanaceous crops (Fiallo-Olive and Navas-Castillo, 2019). The CPm protein encoded by ToCV and other criniviruses was well demonstrated to be associated with virus transmission by vector (Tzanetakis et al., 2013; Kaur et al., 2019) and plausibly possesses the ability to block host RNA silencing for the positive control of the ToCV P22 protein, which is a well-documented RSS protein that does not block RNA silencing (Canizares et al., 2008). Additionally, previous studies demonstrated that the dual activities of pathogenicity and blocking host RNA silencing by functional proteins encoded by plant viruses were also correlated (Peretz et al., 2007). Such is the case that the ToCV CPm protein has been verified to plausibly suppress local ssRNA-induced RNA silencing and to accentuate heterogenous virus replication in plants (Canizares et al., 2008); otherwise, whether to suppress systematic ssRNA- and dsRNA-induced RNA silencing and the molecular mechanisms remain unknown. In this study, the ToCV CPm protein was characterized only to block the local RNA silencing induced by ssRNA and to possess the activity of pathogenicity.

The dual activity of pathogenicity and blocking host RNA silencing was probably not common and conserved in all of the CPm proteins encoded by other criniviruses because the characteristics, including the length, structure, and phylogenetic relationship of CPm proteins, were distinctly different (Figure 1). Further studies of the functional domain(s) and related molecular mechanisms of CPm proteins encoded by each crinivirus would provide deep insight into crinivirus infection.

Usually, plants induce innate defenses *via* RNA silencing immediately after sensing viral infection (Leonetti et al., 2021). ssRNA- and dsRNA-induced RNA silencing, a process referred to as posttranscriptional gene silencing (PTGS), which specifically cleaves viral RNA, is a ubiquitous defense mechanism against RNA viruses (Sheu-Gruttadauria and MacRae, 2017). To counteract this defense response, plant viruses encode functional proteins with the activity of RNA silencing suppression to prevent antiviral RNA silencing by inhibiting different steps of the PTGS pathway (Parent et al., 2012; Li et al., 2015; Hu et al., 2020; Jullien et al., 2020). Multiple examples of RNA silencing suppression systems have been reported in the complex family Closteroviridae (Ambros et al., 2021). The p22 protein, the key RNA silencing suppressor of ToCV, preferentially binds long dsRNAs to prevent its cleavage (Landeo-Rios et al., 2016). The CSR3 protein of SPCSV cleaves double-stranded small interfering RNAs and long dsRNA into fragments that are too short to induce RNA silencing (Weinheimer et al., 2016). The P23 protein of LCV suppressed the onset of local RNA silencing by reducing the accumulation of small-interfering RNA (siRNA) or enhancing the degradation of siRNA (Kubota and Ng, 2016). The CPm protein of ToCV in this study likely employs a novel strategy for direct binding of local small dsRNAs but not ssRNAs to block local ssRNA-induced RNA silencing in *N. benthamiana* (Figures 4, 7). Further characterization of the CPm protein blocking host RNA silencing by ToCV and other criniviruses would contribute to deciphering the interactions of criniviruses with hosts.

The CPm proteins of criniviruses were considered to be present on the end (the ‘tail’) of the virions based on verification from the LIYV CPm protein (Tian et al., 1999). The P22 proteins of criniviruses were not structural proteins and were not present on the virions. It is rational to deduce that the activity of blocking local RNA silencing by the ToCV CPm protein would be indispensable for ToCV primary infection at that point the P22 protein was not translated.

## 5. Conclusion

The results presented here elucidated that the CPm protein encoded by ToCV is a pathogenicity determinant that suppresses host RNA silencing-based antiviral defenses induced by ssRNA but not by dsRNA. The pathogenicity activity of the CPm protein was probably correlated with its activities of accentuating virus replication and triggering the hypersensitive-like response and ER stress in the host. ToCV CPm only suppresses local RNA silencing triggered by single-stranded GFP but not by double-stranded GFP. The activity of blocking host RNA silencing of CPm protein likely resulted in its property of binding dsRNA. The results of this study suggest that blocking host local RNA silencing induced by ssRNA is critical for virus primary infection because the main RNA silencing suppressor of the P22 protein was not translated, which blocks RNA silencing triggered by ss- and dsRNA and induces systematic RNA silencing.

## Data availability statement

The original contributions presented in the study are included in the article/Supplementary material, further inquiries can be directed to the corresponding authors.

## Author contributions

SZ, DZ, and YL contributed to the conception, design of the study, and wrote the manuscript. XY, XWL, YZ, and XYL conducted the study, measured the plant phenotypes, and contributed to laboratory analyses. ZZ, XO, XS, and FL provided the statistical analyses for the manuscript. All authors contributed to the article and approved the submitted version.

## Funding

This work was supported by the National Natural Science Foundation of China (Grant Nos. 32030088, 32072383, and 31972242), China Agricultural Research System (CARS-23-D-02), Talent of Hunan Province (2020TJ-Y05), Innovation Project of Hunan Academy of Agricultural Science (2021CX07).

## Conflict of interest

The authors declare that the research was conducted in the absence of any commercial or financial relationships that could be construed as a potential conflict of interest.

## Publisher’s note

All claims expressed in this article are solely those of the authors and do not necessarily represent those of their affiliated organizations, or those of the publisher, the editors and the reviewers. Any product that may be evaluated in this article, or claim that may be made by its manufacturer, is not guaranteed or endorsed by the publisher.

## Footnotes

^1^ https://www.ncbi.nlm.nih.gov

^2^ https://www.ncbi.nlm.nih.gov/cdd
