# Supplementary material for: Tomato chlorosis virus CPm protein is a pathogenicity determinant and suppresses host local RNA silencing induced by single-stranded RNA
Source: Front Microbiol. 2023 Mar 28;14:1151747. doi: 10.3389/fmicb.2023.1151747 (PMC10086252; doi:10.3389/fmicb.2023.1151747)
Supplement: Supplementary file 1 [file Table_1.DOCX]

**Table S1. The primers and probes used in this study**

| Primer name | Sequence (5’-3’) |
| --- | --- |
| PVX-CPm-F | ATCGATTGGCGCGCCATGATGGATGAAAATGAAATCTATGAG |
| PVX-CPm-R | TCATCGGCGGTCGACGCGGCCGCCTCAGAAAAGCTGACTCGTGCT |
| PVX-CP-qPCR-F | ATGTCAGCACCAGCTAGCACA |
| PVX-CP-qPCR-R | ATCTATCATTTCTGTTTGAGCAGA |
| HSP90-2/qPCR-F  HSP90-2/qPCR-R  bZIP60/qPCR-F  bZIP60/qPCR-R  BLP4/qPCR-F  BLP4/qPCR-R  qActin-F  qActin-R  pBIN-CPm-F  pBIN-CPm-R  CPm-F  CPm-R | AGGGTCAGCTGGAGTTCAA  GGGAAGATCCTCGGAATCCAC  CCTGCTTTGGTTCATGGGCATCAT  AGAAGACCGTGGTTTCTGCTTCGT  AGCTTTGAGCAGTCAACACCAAGT  AAA ACG TGC CCG AGT AAG TGG TTC  TGCCATTCTCCGTCTTGACT  TGCAGTCTCGAGTTCCTGTT  ATTTACGAACGATAGGGTACCATGGATGAAAATGAAATCTATGAG  GTAAGGCCTACTAGTGGATCCTCAGAAAAGCTGACTCGTGCT  CGGGATCCATGGATGAAAATGAAATCTATGAG  CCCTCGAGTCAGAAAAGCTGACTCGTGCT |

Note: The endonuclease site shown by underline.

**Table S2. The probes used for EMSA**

| RNA oligos | Sequence |
| --- | --- |
| siRGFP-guide | 5’-UCAACAGGAUCGAGCUUAAGG-3’ |
| ds siRGFP | 5’-UCAACAGGAUCGAGCUUAAGG-3’  3’-GCAGUUGUCCUAGCUCGAAUU-5’ |
